# Supplementary material for: Resolving the full spectrum of human genome variation using Linked-Reads
Source: Genome Res. 2019 Apr;29(4):635–45. doi: 10.1101/gr.234443.118 (PMC6442396; doi:10.1101/gr.234443.118)
Supplement: Supplemental Material [file supp_gr.234443.118_Supplemental_longranger-2.2.zip › longranger-2.2.tar.bz2!/lr2.2-public/lib/stan/cmdstan/stan/doc/api/html/json_8hpp_source.html]

Stan: src/stan/io/json.hpp Source File


|  |  |
| --- | --- |
|  | Stan  2.5.0  probability, sampling & optimization |


- Main Page
- Related Pages
- Namespaces
- Classes
- Files

- File List
- File Members

All Classes Namespaces Files Functions Variables Typedefs Enumerations Enumerator Friends Macros Pages

- src
- stan
- io

json.hpp

Go to the documentation of this file.

1 #ifndef STAN\_IO\_JSON\_HPP

2 #define STAN\_IO\_JSON\_HPP

3

4 #include "stan/io/json/json\_error.hpp"

5 #include "stan/io/json/json\_handler.hpp"

6 #include "stan/io/json/json\_parser.hpp"

7 #include "stan/io/json/json\_data\_handler.hpp"

8 #include "stan/io/json/json\_data.hpp"

9

10 #endif

json\_parser.hpp

json\_data.hpp

json\_error.hpp

json\_handler.hpp

json\_data\_handler.hpp


---

[ Stan Home Page ]

*© 2011–2014,
Stan Development Team.*
